# Electrodeposited Gold Nanostructures for the Enhancement of Electrochromic Properties of PANI–PEDOT Film Deposited on Transparent Electrode

**DOI:** 10.3390/polym12122778

**Published:** 2020-11-24

**Authors:** Anton Popov, Benediktas Brasiunas, Anzelika Damaskaite, Ieva Plikusiene, Arunas Ramanavicius, Almira Ramanaviciene

**Affiliations:** 1 NanoTechnas—Center of Nanotechnology and Materials Science, Institute of Chemistry, Faculty of Chemistry and Geosciences, Vilnius University, Naugarduko st. 24, LT-03225 Vilnius, Lithuania; anton.popov@chgf.vu.lt (A.P.); benediktas.brasiunas@chgf.vu.lt (B.B.); damaskaite@gmail.com (A.D.); ieva.plikusiene@chgf.vu.lt (I.P.); arunas.ramanavicius@chf.vu.lt (A.R.); 2 Division of Materials Science and Electronics, State Scientific Research Institute Center for Physical Sciences and Technology, Savanorių ave. 231, LT-02300 Vilnius, Lithuania

**Keywords:** electrochromic polymers, gold nanostructures, polyaniline, poly(3,4-ethylenedioxythiophene), conducting polymers

## Abstract

Conjugated polymers (CPs) are attractive materials for use in different areas; nevertheless, the enhancement of electrochromic stability and switching time is still necessary to expand the commercialization of electrochromic devices. To our best knowledge, this is the first study demonstrating the employment of electrodeposited gold nanostructures (AuNS) for the enhancement of CPs’ electrochromic properties when a transparent electrode is used as a substrate. Polyaniline–poly(3,4-ethylenedioxythiophene) (PANI-PEDOT) films were electrodeposited on a transparent indium tin oxide glass electrode, which was pre-modified by two different methods. AuNS were electrodeposited at −0.2 V constant potential for 60 s using both the 1st method (synthesis solution consisted of 3 mM HAuCl_4_ and 0.1 M H_2_SO_4_) and 2nd method (15 mM HAuCl_4_ and 1 M KNO_3_) resulting in an improvement of optical contrast by 3% and 22%, respectively. Additionally, when using the 1st method, the coloration efficiency was improved by 50% while the switching time was reduced by 17%. Furthermore, in both cases, the employment of AuNS resulted in an enhancement of the electrochromic stability of the CPs layer. A further selection of AuNS pre-modification conditions with the aim to control their morphology and size can be a possible stepping stone for the further improvement of CPs electrochromic properties.

## 1. Introduction

Conjugated polymers (CPs) stand out among other electrochromic materials due to their environmental stability, flexibility, biocompatibility, and convenient electrochemical or chemical synthesis [1,2]. Such properties allow CPs to be employed in the design of displays, bioelectronics, smart windows, and electrochromic color-changing textiles [3,4,5,6,7]. However, further investigations of electrochromic materials including CPs are needed to change electrochromic devices such as smart windows from an expensive item to a widely used commodity [8].

A joint utilization of nanostructures with CPs can be the way to improve the desired properties of electrochromic materials. Various nanostructures such as graphene oxide [9], metal oxides [10], MXenes [11], etc. can be used in the fabrication of electrochromic devices. For instance, polyaniline (PANI) layer deposited on indium tin oxide coated glass (ITO) with graphene oxide shows an enhancement of electrochromic stability and improvement of other kinetic parameters [12]. In this context, plasmonic nanostructures are a rather promising candidate for the enhancement of desired CPs properties [13,14]. For instance, the incorporation of metallic nanoparticles into the structure of CPs can be accomplished during the synthesis by the simultaneous reduction of metal salts. Ten nm spherical gold nanoparticles (AuNPs) embedded to electrodeposited nanocomposites of polypyrrole and dye—indigo carmine—enable the enhancement of electrochromic properties such as optical contrast and switching time. In addition, higher electroactivity and lower band-gap energy have been achieved [15]. The incorporation of AuNPs and silver nanoparticles (AgNPs) to the nanocomposite of well-known and widely studied polythiophene derivative poly(3,4-ethylenedioxythiophene) (PEDOT) was performed during oxidative polymerization by Mumtaz et al. [16]. PEDOT–metal particle composites possessed increased coloration efficiency, 3–4 times enhanced contrast ratio, and 4–5 times faster switching between bleached and colored states.

Mixing nanoparticles with polymer solutions is another option of nanocomposite formation. Such a nanocomposite of PEDOT/poly(styrene sulfonate) with 3.2 nm AuNPs or 6.5 nm AgNPs possessed modified colors in colored/bleached states [13]. The incorporation of AuNPs into the structure of PANI copolymerized with *p*-aminothiophenol in the presence of poly(styrene sulfonate) (PSS) was performed by Xiong et al. [17]. Such nanohybrids possessed improved electrochemical activity and shorter switching time. The possibility to control plasmonic resonance by the insertion of gold nanocubes or nanorods to an electrochromic polymer matrix was shown previously [18,19]. An alternative approach is based on plasmonic nanostructures that are deposited on the surface. The usage of gold nanomesh structures as substrate allows for the moderation of a deposited thin PANI layer color under electrochemical switching [20]. The electrochromic polymer layer color change was monitored where an Au film with etched nanoholes [21], and Au or Al nanoslit arrays [22], were used as substrates for polymer deposition. Furthermore, a PANI layer on a metallic nanoslit exhibits faster electrochromic switching, and its color can be controlled by changing slit dimensions.

Gold nanostructures (AuNS) can be electrodeposited on the surface of various electrodes [23], wherein the morphology of deposited AuNS could be easily controlled to some extent by choosing deposition conditions, such as synthesis time, electrode potential, and the composition of the synthesis solution [24]. It is well known that optical and electrochemical properties depend on the size and morphology of AuNS [25]. According to previously discussed articles, various AuNS affect the optical and electrochromic properties of CPs differently. It can be assumed that the interaction between CPs and AuNS may vary depending on the size and morphology of AuNS.

The enhancement of electrochromic performance and stability is needed for a wider application of CPs in different areas such as smart windows and display devices [26]. Plasmonic nanostructures, which negligibly affect substrate transparency and have a positive impact on CPs’ electrochromic properties, are a potentially interesting object to study.

In this work, we investigated how plasmonic nanostructures affect the electrochromic properties of CPs. To our knowledge, the effects of AuNS deposited on a transparent electrode were investigated for the first time. AuNS were synthesized on an ITO electrode and polyaniline-poly(3,4-ethylenedioxythiophene) (PANI-PEDOT) films were electrodeposited on top of ITO/AuNS substrate.

## 2. Experimental

### 2.1. Materials and Methods

3,4-Ethylenedioxythiophene (EDOT), tetrachloroauric acid trihydrate (HAuCl_4_·3H_2_O), and ITO (15–25 Ω cm^−1^) were purchased from Sigma-Aldrich (Steinheim, Germany). Aniline (ANI) and sodium dihydrogen phosphate monohydrate were bought from Fluka (Buchs, Switzerland). Sulfuric acid was obtained from Roth (Karlsruhe, Germany). Potassium chloride were purchased from Merck. Lithium perchlorate and acetone were bought from Alfa Aesar (Karlsruhe, Germany). Aniline was distilled once before use. All aqueous solutions were prepared with deionized water (18 MΩ·cm^−1^).

### 2.2. Pre-Treatment of ITO Electrode

All electrochemical measurements were performed using a three-electrode cell and a potentiostat PGSTAT30/Autolab from ECOChemie (Utrecht, Netherlands) with GPES 4.9 software. An ITO electrode, which was cut to required size rectangles, was used as a working electrode. A platinum electrode served as a counter electrode and an Ag/AgCl_(3M KCl)_ electrode from CH Instruments (Austin, TX, USA) was used as a reference. Before synthesis, the ITO electrode was washed with acetone and then sequentially treated by ultrasound for 15 min each in acetone and water. Additionally, the ITO electrode was cleaned by potential cycling between 0 and +1 V in 50 mM phosphate-buffered saline (PBS), pH 6, with 0.1 M KCl and then rinsed with deionized water.

### 2.3. Electrodeposition of Gold Nanostructures

AuNS were electrodeposited at −0.2 V constant potential for 60 s. Two aqueous synthesis solutions were used for the formation of AuNS differing in morphology and shape. For the 1st synthesis method, the solution from which AuNS_I_ were formed consisted of 3 mM HAuCl_4_ and 0.1 M H_2_SO_4_, while the 2nd synthesis method solution consisted of 15 mM HAuCl_4_ and 1 M KNO_3_ (AuNS_II_). After AuNS were formed on an ITO electrode, the layer was carefully washed with deionized water and left to dry in air.

### 2.4. Preparation of Polymer Films

PANI-PEDOT films were electrodeposited using the cyclic voltammetry method with a three-electrode electrochemical cell by our previously reported method [3]. Electrodeposition was performed from a water-based solution consisting of 0.2 M ANI, 0.01 M EDOT, 0.2 M H_2_SO_4_, and 0.1 M LiClO_4_ as the supporting electrolyte. The voltage was swept between 0 and +1.1 V with a potential sweep rate of 50 mV s^−1^ for 10 cycles. After electrodeposition, synthesized polymer films were gently rinsed with deionized water to remove the oligomers and inorganic salt from the surface of the polymer layer.

### 2.5. Characterization of AuNS and PANI-PEDOT Films

An electrochemically active area of formed AuNS was determined for both synthesis methods using the cyclic voltamperometry method where the ITO/AuNS electrode was submerged in 0.5 M H_2_SO_4_ solution and the potential was cycled between 0 and +1.4 V at the potential sweep rate of 50, 100, and 150 mV s^−1^. The electrochemically active area (*Γ*) was calculated using this equation [27]:

(1) $$\Gamma =\frac{A}{\nu \cdot 400\;\mu C\;{\mathrm{cm}}^{-1}}$$

where *A*—the area of the cathodic current, *ν*—the potential sweep rate, and 400 μC cm^−1^—the charge density per unit area associated with the electrochemical reduction of a monolayer of chemisorbed oxygen on polycrystalline gold [28].

The color of electrodeposited AuNS was evaluated using a Flame spectrometer with a tungsten halogen light source HL 2000 (OceanOptics, Dunedin, FL, USA). A white standard WS-1-SL and reflection probe QR400-7-VIS-NIR were used for calibrating the flame spectrometer before measurements. The CIELAB 1976 color space coordinates [29] were selected for the evaluation of the ITO/AuNS sample color.

The polycrystallinity of AuNS was determined by performing the X-ray diffraction method (XRD) using MiniFlex II diffractometer (Rigaku, Tokyo, Japan). XRD was performed in the angle range of 10° to 80° with a rate of 5° per minute. The shape of gold nanostructures for both synthesis methods and the morphology of PANI/PEDOT films deposited on ITO/AuNS were analyzed using scanning electron microscope SU-70 (SEM) (Hitachi, Krefeld, Germany).

The absorbance spectra of AuNS and PANI-PEDOT films were determined using a UV-Vis spectrometer Lambda 25 (Perkin Elmer, Waltham, MA, USA) and were registered in the range from 400 to 1100 nm. The thickness of the polymer layers was evaluated by atomic force microscope BioScope Catalyst (Bruker, Billerica, MA, USA). A gold-coated silicon nitride cantilever (spring constant 0.06 N m^−1^, resonant frequency 24 kHz) was used. AFM measurements were performed in contact mode at the junction of the polymer and ITO left after scratching polymer layers with a soft plastic stick.

### 2.6. Electrochromic Switching

Measurements of electrochromic performance of PANI-PEDOT layers were performed using the same potentiostat and 3-electrode system, which was used for AuNS and polymers deposition. Potential was cycled between −0.1 and +0.5 V, while the absorbance of PANI-PEDOT layers was registered at the same time. PANI-PEDOT layers deposited on a bare and AuNS-coated ITO electrode were compared using an absorbance value at *λ*_max_ of the polymer’s colored state.

## 3. Results

**Figure 1:**
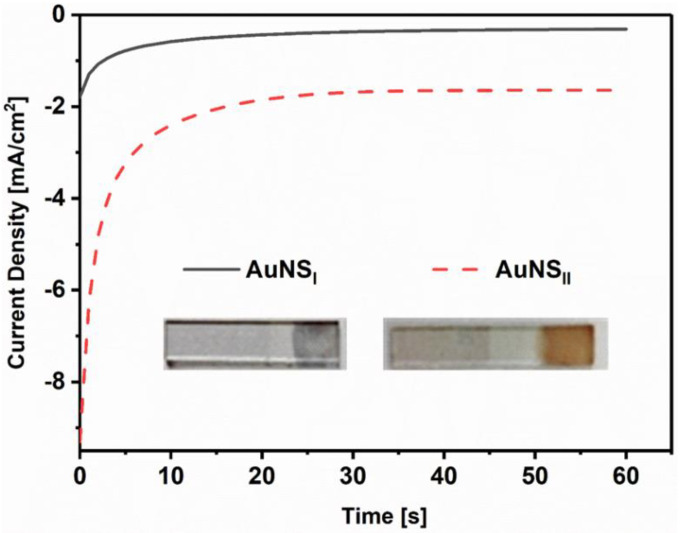
Electrochemical deposition of gold nanostructures (AuNS) on an indium tin oxide (ITO) electrode. Inset: Photographic pictures of ITO/AuNS electrodes after electrodeposition from different solutions.

AuNS with different shapes and morphology were electrodeposited onto a pre-cleaned ITO electrode using two methods. During the 1st synthesis method, deposition was performed from a water-based solution consisting of 3 mM HAuCl_4_ and 0.1 M H_2_SO_4_ (AuNS_I_), while the 2nd method used 15 mM HAuCl_4_ and 1 M KNO_3_ (AuNS_II_). In both cases, electrochemical deposition was performed at a constant −0.2 V working electrode potential vs. Ag/AgCl_(3M KCl)_ reference electrode for 60 s. The chronoamperograms registered during electrochemical deposition are presented in Figure 1. During the synthesis, there was a clear visual indication that AuNS were depositing on ITO because the color of the formed AuNS layer was becoming more intensive with time using both AuNS formation methods. After the synthesis, colors of AuNS_I_ and AuNS_II_ were light gray (L* = 47.2, a* = −2.1, b* = 10.3) and intense dark-orange (L* = 50.6, a* = 20.0, b* = 20.6), respectively.

**Figure 2:**
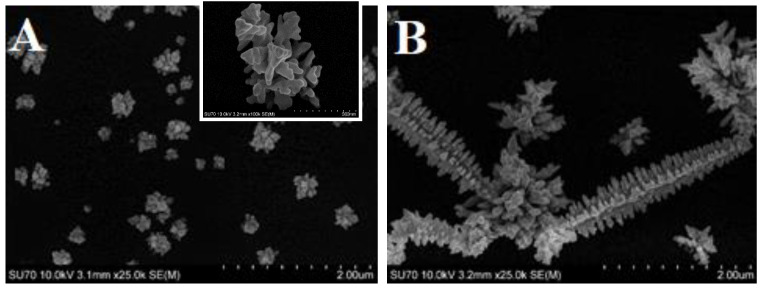
SEM images of (**A**) ITO/AuNS_I_ and (**B**) ITO/AuNS_II_ deposited at −0.2 V for 60 s from a water-based solutions.

The morphology of synthesized AuNS was evaluated using the SEM imaging technique (Figure 2). Using the 1st method, anisotropic “hedgehog” shape AuNS, which were around 200–500 nm in size, were deposited on an ITO electrode. AuNS were located randomly at various distances apart from one another. On the contrary, significantly larger dendritic microstructures with branches were observed after AuNS synthesis using the 2nd method. Higher HAuCl_4_ concentration in the case of the 2nd method possibly had an impact on the formation of larger AuNS. Similar structures were registered when AuNS were electrodeposited on an ITO electrode by applying −0.3 V for 3600 s [30]. It is well known that the morphology and shape of AuNS depend on the applied potential, deposition time, and the composition of the synthesis solution [24]. In our case, the reason for such a difference in AuNS morphology possibly lies in the choice of supporting electrolyte and the pH value of the initial solution. On the other hand, initial HAuCl_4_ concentration is likely to influence the size of the AuNS rather than the morphology. The replacement of Cl^−^ ligands by OH^−^ groups in AuCl_4_^−^ ion tends to decrease the nucleation rate and since the gold complex degree of hydrolysis depends on the pH, more reactive Au complexes are present in acidic solutions. In turn, this leads to the formation of a larger number of synthesis nuclei at acidic conditions [31]. Therefore, as expected, a larger number of smaller AuNS were deposited using the 1st method.

**Figure 3:**
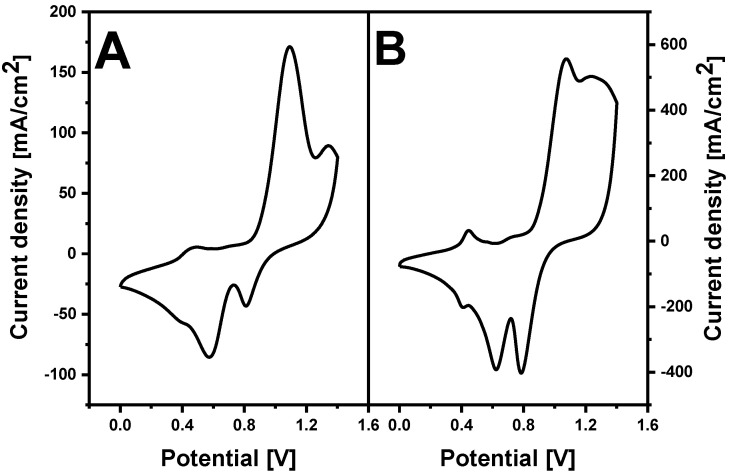
Cyclic voltammograms of (**A**) ITO/AuNS_I_ and (**B**) ITO/AuNS_II_ recorded in 0.5 M H_2_SO_4_ solution. The potential sweep rate was 150 mV s^−1^.

Oxygen adsorption measurements were chosen to determine the electroactive surface area of synthesized AuNS [32]. Figure 3 represents ITO/AuNS cyclic voltammograms collected in 0.5 M H_2_SO_4_ solution. Electrochemical gold oxide formation and subsequent reduction were observed. The electroactive surface area was found to be 0.19 ± 0.03 cm^2^ and 1.44 ± 0.17 cm^2^ for AuNS synthesized using the 1st and 2nd method respectively.

**Figure 4:**
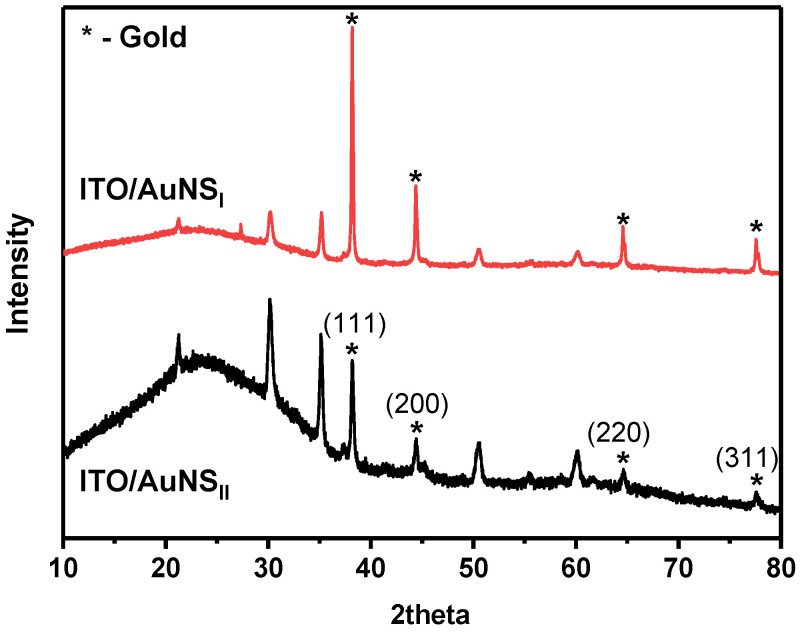
XRD patterns of AuNS deposited on an ITO electrode.

Cyclic voltammograms also displayed a broad region of gold oxide reduction. In both cases, three oxidation and three reduction peaks were registered. This fact reveals the polycrystalline nature of synthesized AuNS [33]. The position of oxidation/reduction peaks depends on the crystallographic orientation of the gold surface [34]. The oxidation peak observed with both methods at around +1.3 V is attributed to the Au (111) plane [35]. Additional XRD measurements were performed for the determination of the AuNS crystalline structure (Figure 4). The results clearly indicate that the formed AuNS are polycrystalline. All patterns have the lines of Au(111), Au(200), Au(220), and Au(311) crystal faces. It illustrates the fcc crystalline structure of AuNS [35]. In both cases, the intensity of the Au(111) diffraction peak was much stronger when compared to others. The ratio of gold and ITO facets intensities was higher for ITO/AuNS_II_. These results coincide with CV measurements and indicate that a higher amount of gold was deposited using the 2nd method.

**Figure 5:**
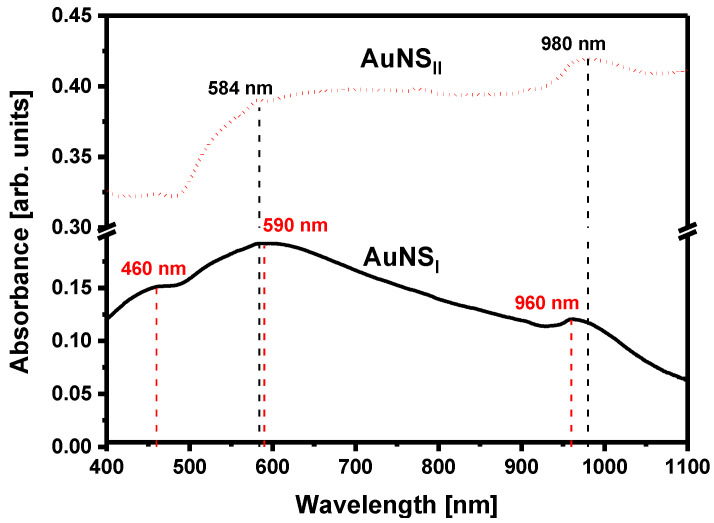
Absorption spectra of AuNS deposited on an ITO electrode.

Since electrodeposition was performed on a transparent ITO electrode, the optical properties of AuNS could be conveniently studied using the UV-Vis-NIR spectroscopy method. Absorption spectra were recorded in the range from 400 to 1100 nm (Figure 5). In the absorption spectra of AuNS_I_, three maxima at 460, 590, and 960 nm were registered. In the case of AuNS_II_, only two peaks at 584 nm and 980 nm were observed. The broad peaks at 960 nm for AuNS_I_ and at 980 nm for AuNS_II_ are present due to in-plane dipole resonance, whereas peaks at visible parts of the spectrum are attributed to the out-of-plane dipole resonance [36]. In comparison, surface plasmon resonance (SPR) peaks at 550 nm and at about 940 nm were registered in the spectra of Au nanoplates synthesized on an ITO electrode [37,38]. Differences in the absorbance intensity of AuNS and the position of *λ*_max_ can be explained by the distinctions in shape, morphology, aspect ratio, and sizes of AuNS synthesized using different methods [39,40]. For instance, local roughness can affect the AuNS optical properties [41]. In addition, the broad absorption throughout the whole visible spectra can likely be attributed to an uneven AuNS size on the surface, resulting in continuous absorption and diffraction at various wavelengths. Furthermore, our results showed that the active surface area of AuNS synthesized using the 2nd method was much larger, which possibly represents the differences in AuNS absorbance intensity between synthesis methods.

**Figure 6:**
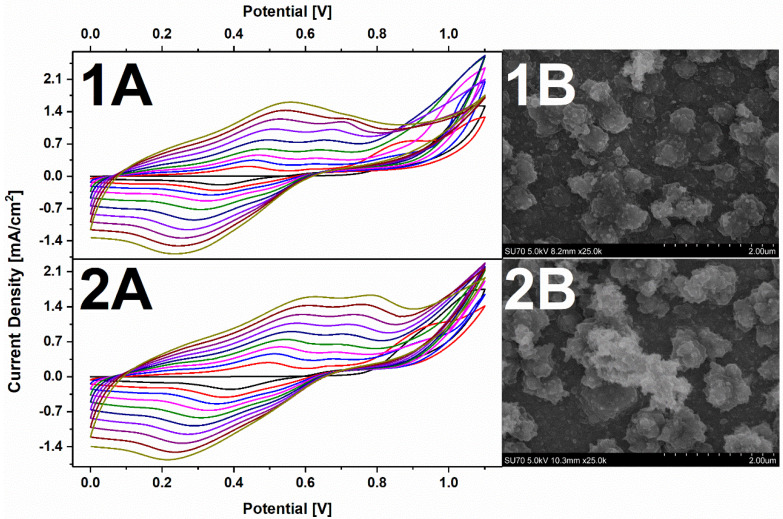
(**A**) Cyclic voltammograms obtained during the synthesis and (**B**) SEM images of polyaniline–poly(3,4-ethylenedioxythiophene) (PANI-PEDOT) films on (**1**) ITO/AuNS_I_ and (**2**) ITO/AuNS_II_ electrodes. Electrochemical synthesis was performed in 0.2 M H_2_SO_4_ solution containing 0.1 M LiClO_4_, the sweep rate of the electrode potential was 50 mV s^−1^.

PANI-PEDOT layers were electrochemically deposited on the surface of ITO/AuNS_I_ and ITO/AuNS_II_ substrates (Figure 6). Moreover, the synthesis of the polymer layer was also performed on the surface of a bare ITO electrode without AuNS to be used as a control sample. Typical cyclic voltammograms were monitored during PANI-PEDOT electrodeposition on ITO/AuNS [3]. The formation of a “nucleation loop” during the first few cycles can be associated with the reaction between PANI and PEDOT monomers and the formation of their oligomers [42]. PANI polymerization is confirmed by the presence of oxidation peaks at +0.55 V and +0.75 V in the case of electrodeposition on ITO/AuNS_I_ and at +0.61 V and +0.80 V in the case of an ITO/AuNS_II_ substrate. The first peak in both cases represents the formation of breakdown products such as p-benzoquinone and hydroquinone [43]. Oxidation peaks at +0.75 V and +0.80 V are related to the formation of a PANI polymerization chain by the generation of diradical–dications, while at potential higher than +0.80 V, an interaction of diradical–dications and PANI monomers takes place [44]. It is also worth noting that PEDOT polymerization occurs in the range from +1 to +1.1 V [45]. According to our previous work [3], a PANI and PEDOT composite is formed during polymerization, since the formation of aniline radicals and EDOT polymerization take place at significantly different potentials.

The morphology of the synthesized PANI-PEDOT layers did not change regardless of the method used for AuNS formation (Figure 6). Moreover, the surface of the electrodeposited nanocomposite was similar to the surface of the PANI-PEDOT layer deposited on the bare ITO electrode. Observed globular structures are attributed to the PEDOT polymer, wherein lump-like and fibrous clumps are characteristic features of PANI structures [3].

**Figure 7:**
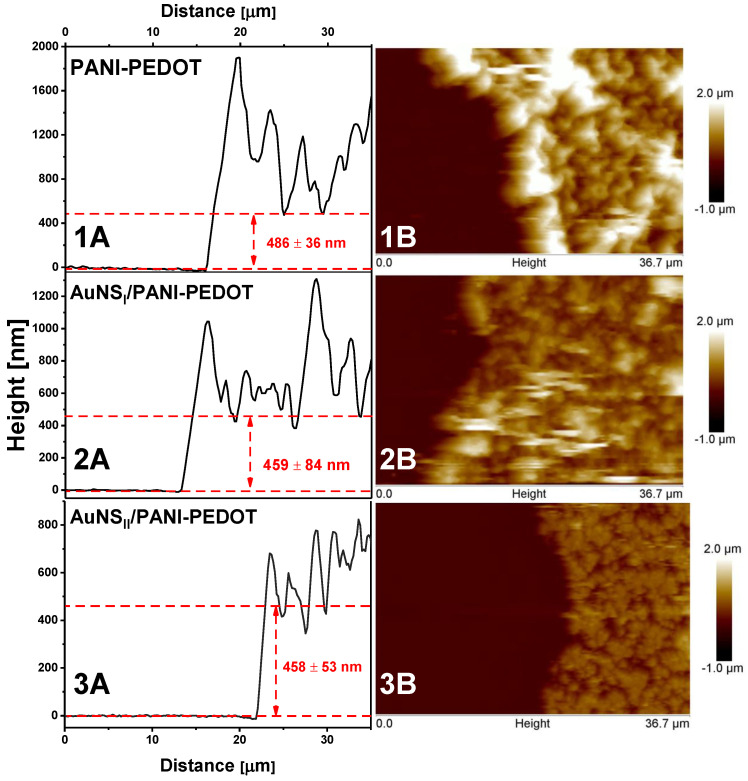
The surface topography evaluation of a PANI-PEDOT layer deposited on (**1**) ITO, (**2**) ITO/AuNS_I_, and (**3**) ITO/AuNS_II_ obtained by AFM. The surface section (**A**) and 2D AFM image (**B**) are presented.

Since the deposition of PANI-PEDOT layers was performed under the same conditions for the bare ITO electrode and ITO/AuNS electrodes, it was theorized that the thickness of the polymer layers will be the same regardless of the increase in surface area observed due to the formation of AuNS, since the growth after a first few electrodeposition cycles continues on the freshly formed polymer layer. AFM measurements in contact mode (Figure 7) were performed to confirm this statement.

Morphology typical for electrodeposited polymers was determined. A large number of irregularities were present in all cases on the PANI-PEDOT surface, resulting in relatively rough polymer layers. The layer thickness was similar for all different substrates. For bare ITO, ITO/AuNS_I_, and ITO/AuNS_II_, it was equal to 486 ± 36 nm, 459 ± 84 nm, and 458 ± 53 nm, respectively. The same effect was observed in the literature where the PANI layer was electrochemically deposited on a flat gold surface and gold nanomesh substrates [20].

**Figure 8:**
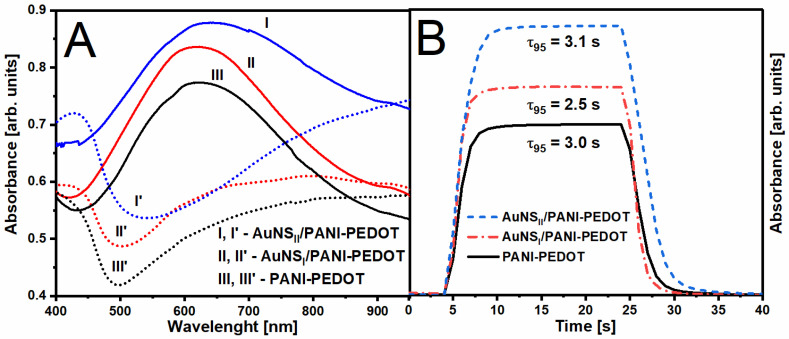
(**A**) Absorbance spectra of the PANI-PEDOT layer deposited on different substrates under (**dotted lines**) −0.1 V and (**solid lines**) +0.5 V applied potential. (**B**) Absorbance at *λ*_max_ dependency on time during electrochromic switching between −0.1 and +0.5 V potential (measurements were done in 0.2 M H_2_SO_4_ and 0.1 M LiClO_4_ solution when potential was applied vs. Ag/AgCl electrode).

The electrochromic performance of the prepared polymer layers was investigated by the spectrochronoamperometry method. Primarily, absorbance spectra were registered after applying −0.1 or +0.5 V potential to the polymer layers (Figure 8A). After applying +0.5 V (colored state), a slight shift in the position of absorption maximum from 623 to 628 nm was observed in the case of the PANI-PEDOT layer deposited on the AuNS_II_ substrate compared with other substrates. A similar effect for the absorption minimum was monitored while in a bleached state (−0.1 V) wherein a more significant *λ*_min_ red shift from 501 to 541 nm was detected. A similar large red shift of absorbance maximum in the colored state was reported by Shahabuddin et al. where the PANI layer was deposited on nanomesh gold structures [20]. Those results were obtained in reflection mode, and the red shift was observed in comparison with the PANI layer deposited on a flat gold surface. It clearly illustrates that such an effect is achieved due to the presence of gold nanostructures. However, in our case, only AuNS_II_ resulted in such a shift. It could possibly be explained by the different interaction between polymers and AuNS.

Switching time (*τ_95_*) is an important kinetic parameter for comparing electrochromic materials. In this study, the response was calculated as the time that is needed for an optical change of 95% to happen between colored and bleached states (Figure 8B). The optical change was monitored at *λ*_max_, which was observed in the colored state (+0.5 V) of the PANI-PEDOT layers. In the case of the ITO/AuNS_II_ substrate, the switching time slightly increases from 3.0 to 3.1 s when compared to PANI-PEDOT on a bare ITO electrode, wherein *τ_95_* using AuNS_I_ on the contrary decreases to 2.5 s. A comparable reduction of switching time was obtained after the incorporation of gold nanoparticles to the nanocomposite of indigo carmine-doped polypyrrole [15]. The usage of AuNS in both cases led not only to the increase of background absorbance (Figure 8A) but also to the improvement of optical contrast (Δ*T*) (Table 1). This difference can be associated with the variation in size and surface concentration of gold nanostructures.

**Figure 9:**
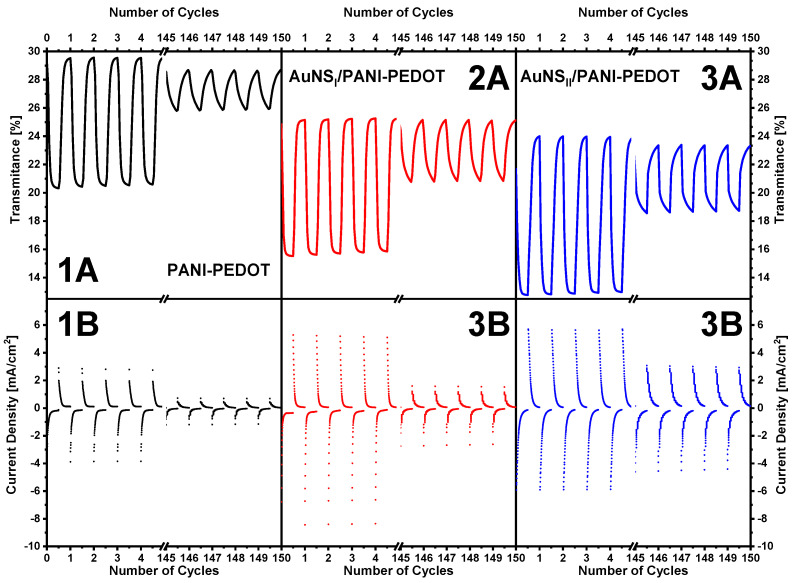
(**A**) Transmittance curves at *λ*_max_ and (**B**) chronoamperometry results of PANI-PEDOT layers synthesized on (**1**) ITO, (**2**) ITO/AuNS_I_ and (**3**) ITO/AuNS_II_ registered in 0.2 M H_2_SO_4_ with 0.1 M LiClO_4_ solution. First 5 and last 146–150 cycles are displayed. The potential was switched between −0.1 V and +0.5 V vs. Ag/AgCl for 10 s at each step.

Additional experiments were done for the evaluation of the influence of AuNS on polymer’s electrochromic performance and cycling stability. Electrochromic switching between −0.1 and +0.5 V was performed for 150 cycles (Figure 9). Optical change was monitored at the same wavelengths as were used in the previous experiment. Coloration efficiency (*CE*) was calculated according to the following Equations [46]:

(2) $$\Delta QD=log\left(\frac{T_{b}}{T_{c}}\right)\;\;\;\;\;\;\;\;\;\;\;\;\;\;\;\;\;\;\;\;\;\;\;\;\;\;\;\;\;\;\;\;\;\;CE=\;\frac{\Delta QD}{Q}$$

where Δ*QD* is optical density change, which was calculated using transmittance values of polymer’s bleached (*T_b_*) and colored (*T_c_*) states, and *Q* is the electronic charge consumed per unit area.

It can obviously be seen that AuNS affect the electrochromic performance of the PANI-PEDOT layer. The addition of AuNS_I_ to the system results in an increase of *CE* value from 21.7 to 32.8 cm^2^ C^−1^. The value of Δ*T* improves from 8.7% to 9.0% when compared with PANI-PEDOT on a bare ITO electrode. On the other hand, optical contrast was higher (10.6%) when AuNS_II_ were used; however, the calculated *CE* value was practically the same compared with PANI-PEDOT on a bare ITO electrode. These results go hand in hand with the data from switching time measurements, with AuNS_I_ providing the best results. A minor decrease in the transmittance of the investigated layers is monitored due to the AuNS interaction with light (absorbance, diffraction, etc.). Additionally, samples with AuNS showed better electrochromic stability. After 150 cycles of electrochromic switching, the Δ*T* value of the PANI-PEDOT layer deposited on the bare ITO electrode decreases about 1.5 times more in comparison with that determined for the polymer layers deposited on ITO/AuNS, wherein *CE* values decrease relatively equally. The observable and expected decrease of optical contrast is associated with dopants leaching out of the polymer during electrochromic switching accompanied by changes in structure and conformation due to the accumulation of extra charges in the chains [15].

The deposition of plasmonic nanostructures typically allows for the increase of electrode surface area, thereby possibly affecting charge transport kinetics due to interface effects [20,47]. These effects are related to a quite low work function value for AuNS. For instance, gold nanoparticles with values of 3.4 eV and 3.6 eV were recently described in the literature [48,49]. In our case, it is important to pay attention to the work function value of ITO being approximately equal to 4.5 eV [50], while that of PANI is 4.42 eV [51], and PEDOT in the form of a mixture with PSS has been reported from 4.7 to 5.4 eV [52]. Therefore, the change in electrochromic properties due to the formation of the depletion layer and Schottky barrier in polymers, which are p-type semiconductors, is expected [20]. Different effects on electrochromic properties can be associated with a distinct work function for particular gold nanostructures. Previously, it was shown that the work function [53] and electron affinity [54] of gold clusters depend on their size.

## 4. Conclusions

The enhancement of the electrochromic properties of the PANI-PEDOT layer, which was provided by AuNS deposited on an ITO electrode, has been observed. Such an easy and low-cost method of substrate pre-modification can be used for further improvements of various electrochromic devices based on conducting polymers. This research demonstrated that the electrochemical and optical properties of AuNS can be controlled by changing electrodeposition parameters. We believe that future investigations will allow for the selection of AuNS synthesis conditions that result in better optical transmittance while possessing enough significant positive effects on the electrochromic properties of conducting polymers. In addition, the possibility to increase the optical contrast and to control the color of the CPs layer could reduce the need for combining several CPs to achieve a full-color gamut during the fabrication of electrochromic devices. Moreover, the electrodeposition of other plasmonic nanostructured materials can potentially be used to tune the electrochromic properties of CPs. Thus, the use of transparent substrates with nanostructures can allow extending possible areas of CPs’ application in smart windows and flexible electrochromic displays.

## Figures and Tables

**Table 1:** Electrochromic properties of the PANI-PEDOT layer deposited on different substrates.

Substrate	*N*	*Q* [mC cm^−2^]	*T_c_*/*T_b_* [%]	Δ*T* [%]	*CE* [cm^2^ C^−1^]
**ITO**	1	5.6	29.4/20.7	8.7	21.7
	150	2.3	28.6/26.0	2.6	14.3
**ITO/AuNS_I_**	1	4.8	24.9/15.9	9.0	32.8
	150	2.5	25.1/21.0	4.1	24.5
**ITO/AuNS_II_**	1	10.0	23.8/13.2	10.6	20.5
	150	3.8	23.5/18.8	4.7	14.5

## References

[1] Spychalska K., Zajac D., Baluta S., Halicka K., Cabaj J. (2020). Functional polymers structures for (Bio)sensing application—A review. Polymers.

[2] Deshmukh M.A., Gicevicius M., Ramanaviciene A., Shirsat M.D., Viter R., Ramanavicius A. (2017). Hybrid electrochemical/electrochromic Cu(II) ion sensor prototype based on PANI/ITO-electrode. Sens. Actuators B Chem..

[3] Popov A., Brasiunas B., Mikoliunaite L., Bagdziunas G., Ramanavicius A., Ramanaviciene A. (2019). Comparative study of polyaniline (PANI), poly(3,4-ethylenedioxythiophene) (PEDOT) and PANI-PEDOT films electrochemically deposited on transparent indium thin oxide based electrodes. Polymer.

[4] Mantione D., del Agua I., Sanchez-Sanchez A., Mecerreyes D. (2017). Poly(3,4-ethylenedioxythiophene) (PEDOT) derivatives: Innovative conductive polymers for bioelectronics. Polymers.

[5] Le T.-H., Kim Y., Yoon H. (2017). Electrical and electrochemical properties of conducting polymers. Polymers.

[6] Celiesiute R., Ramanaviciene A., Gicevicius M., Ramanavicius A. (2019). Electrochromic sensors based on conducting polymers, metal oxides, and coordination complexes. Crit. Rev. Anal. Chem..

[7] Wang H., Yao C.-J., Nie H.-J., Yang L., Mei S., Zhang Q. (2020). Recent progress in integrated functional electrochromic energy storage devices. J. Mater. Chem. C.

[8] Kraft A. (2019). Electrochromism: A fascinating branch of electrochemistry. ChemTexts.

[9] Shi Y., Zhang Y., Tang K., Song Y., Cui J., Shu X., Wang Y., Liu J., Wu Y. (2018). In situ growth of PEDOT/graphene oxide nanostructures with enhanced electrochromic performance. RSC Adv..

[10] Zheng J., Chen L., Liu S., Sun C., Hu X., Zhou S. (2019). MoO3@PEDOT coaxial heterostructure nanobelts by in situ polymerization with enhanced electrochromic performance. Mater. Res. Express.

[11] Li J., Levitt A., Kurra N., Juan K., Noriega N., Xiao X., Wang X., Wang H., Alshareef H.N., Gogotsi Y. (2019). MXene-conducting polymer electrochromic microsupercapacitors. Energy Storage Mater..

[12] Zhao L., Zhao L., Xu Y., Qiu T., Zhi L., Shi G. (2009). Polyaniline electrochromic devices with transparent graphene electrodes. Electrochim. Acta.

[13] Pacios R., Marcilla R., Pozo-Gonzalo C., Pomposo J.A., Grande H., Aizpurua J., Mecerreyes D. (2007). Combined electrochromic and plasmonic optical responses in conducting polymer/metal nanoparticle films. J. Nanosci. Nanotechnol..

[14] Mikoliunaite L., Kubiliute R., Popov A., Voronovič J., Šakirzanovas S., Ramanavičiene A., Ramanavičius A. (2014). Development of gold nanoparticle-polypyrrole nanocomposites. Chemija.

[15] Loguercio L.F., Alves C.C., Thesing A., Ferreira J. (2015). Enhanced electrochromic properties of a polypyrrole-indigo carmine-gold nanoparticles nanocomposite. Phys. Chem. Chem. Phys..

[16] Mumtaz M., Ouvrard B., Maillaud L., Labrugere C., Cloutet E., Cramail H., Delville M.-H. (2012). Hybrid PEDOT-metal nanoparticles—New substitutes for PEDOT:PSS in electrochromic layers—Towards improved performance. Eur. J. Inorg. Chem..

[17] Xiong S., Lan J., Yin S., Wang Y., Kong Z., Gong M., Wu B., Chu J., Wang X., Zhang R. (2018). Enhancing the electrochromic properties of polyaniline via coordinate bond tethering the polyaniline with gold colloids. Sol. Energy Mater. Sol. Cells.

[18] König T.A.F., Ledin P.A., Kerszulis J., Mahmoud M.A., El-Sayed M.A., Reynolds J.R., Tsukruk V.V. (2014). Electrically tunable plasmonic behavior of nanocube-polymer nanomaterials induced by a redox-active electrochromic polymer. ACS Nano.

[19] Ledin P.A., Jeon J.W., Geldmeier J.A., Ponder J.F., Mahmoud M.A., El-Sayed M., Reynolds J.R., Tsukruk V.V. (2016). Design of hybrid electrochromic materials with large electrical modulation of plasmonic resonances. ACS Appl. Mater. Interfaces.

[20] Shahabuddin M., McDowell T., Bonner C.E., Noginova N. (2019). Enhancement of electrochromic polymer switching in plasmonic nanostructured environment. ACS Appl. Nano Mater..

[21] Zhang S., Yu S., Zhou J., Ponder J.F., Smith M.J., Reynolds J.R., Tsukruk V.V. (2019). Heterogeneous forward and backward scattering modulation by polymer-infused plasmonic nanohole arrays. J. Mater. Chem. C.

[22] Xu T., Walter E.C., Agrawal A., Bohn C., Velmurugan J., Zhu W., Lezec H.J., Talin A.A. (2016). High-contrast and fast electrochromic switching enabled by plasmonics. Nat. Commun..

[23] Feng L., Niu M., Wen Z., Hao X. (2018). Recent advances of plasmonic organic solar cells: Photophysical investigations. Polymers.

[24] Terao K., Kakita C., Nagase N., Miyanishi N., Suzuki T., Takao H., Shimokawa F., Oohira F. (2012). Evaluation of electrodeposited gold nanostructures for applications in QCM sensing. Anal. Sci..

[25] Li Y., Shi G. (2005). Electrochemical growth of two-dimensional gold nanostructures on a thin polypyrrole film modified ITO electrode. J. Phys. Chem. B.

[26] Yu F., Liu W., Ke S.-W., Kurmoo M., Zuo J.-L., Zhang Q. (2020). Electrochromic two-dimensional covalent organic framework with a reversible dark-to-transparent switch. Nat. Commun..

[27] Nikolaev K., Ermakov S., Ermolenko Y., Averyaskina E., Offenhäusser A., Mourzina Y. (2015). A novel bioelectrochemical interface based on in situ synthesis of gold nanostructures on electrode surfaces and surface activation by Meerwein’s salt. A bioelectrochemical sensor for glucose determination. Bioelectrochemistry.

[28] Trasatti S., Petrii O.A. (1992). Real surface area measurements in electrochemistry. J. Electroanal. Chem..

[29] Stojkoski V., Kert M. (2020). Design of pH responsive textile as a sensor material for acid rain. Polymers.

[30] Shu H., Cao L., Chang G., He H., Zhang Y., He Y. (2014). Direct electrodeposition of gold nanostructures onto glassy carbon electrodes for non-enzymatic detection of glucose. Electrochim. Acta.

[31] Tran M., Mundt C., Lan T., Padalkar S. (2017). Electrodeposition of gold nanostructures having controlled morphology. J. Nanosci. Nanotechnol..

[32] Oesch U., Janata J. (1983). Electrochemical study of gold electrodes with anodic oxide films-I. Formation and reduction behaviour of anodic oxides on gold. Electrochim. Acta.

[33] Plowman B.J., O’Mullane A.P., Bhargava S.K. (2011). The active site behaviour of electrochemically synthesised gold nanomaterials. Faraday Discuss..

[34] Hamelin A. (1996). Cyclic voltammetry at gold single-crystal surfaces. Part 1. Behaviour at low-index faces. J. Electroanal. Chem..

[35] Tian Y., Liu H., Zhao G., Tatsuma T. (2006). Shape-controlled electrodeposition of gold nanostructures. J. Phys. Chem. B.

[36] Millstone J.E., Park S., Shuford K.L., Qin L., Schatz G.C., Mirkin C.A. (2005). Observation of a quadrupole plasmon mode for a colloidal solution of gold nanoprisms. J. Am. Chem. Soc..

[37] Sajanlal P.R., Pradeep T. (2008). Growth of anisotropic gold nanostructures on conducting glass surfaces. J. Chem. Sci..

[38] Goy-López S., Castro E., Taboada P., Mosquera V. (2008). Block copolymer-mediated synthesis of size-tunable gold nanospheres and nanoplates. Langmuir.

[39] Yu K., Kelly K.L., Sakai N., Tatsuma T. (2008). Morphologies and surface plasmon resonance properties of monodisperse bumpy gold nanoparticles. Langmuir.

[40] Yu Y.Y., Chang S.S., Lee C.L., Wang C.R.C. (1997). Gold nanorods: Electrochemical synthesis and optical properties. J. Phys. Chem. B.

[41] Wang H., Goodrich G.P., Tam F., Oubre C., Nordlander P., Halas N.J. (2005). Controlled texturing modifies the surface topography and plasmonic properties of Au nanoshells. J. Phys. Chem. B.

[42] Zhou C., Liu Z., Du X., Ringer S.P. (2010). Electrodeposited PEDOT films on ITO with a flower-like hierarchical structure. Synth. Met..

[43] Mirmohseni A., Wallace G. (2003). Preparation and characterization of processable electroactive polyaniline–polyvinyl alcohol composite. Polymer.

[44] Chen W.-C., Wen T.-C., Gopalan A. (2002). Negative capacitance for polyaniline: An analysis via electrochemical impedance spectroscopy. Synth. Met..

[45] Sakmeche N., Aeiyach S., Aaron J.-J., Jouini M., Lacroix J.C., Lacaze P.-C. (1999). Improvement of the electrosynthesis and physicochemical properties of poly(3,4-ethylenedioxythiophene) using a sodium dodecyl sulfate micellar aqueous medium. Langmuir.

[46] Yue H., Kong L., Wang B., Yuan Q., Zhang Y., Du H., Dong Y., Zhao J. (2019). Synthesis and characterization of novel D-A type neutral blue electrochromic polymers containing pyrrole[3-c]pyrrole-1,4-diketone as the acceptor units and the aromatics donor units with different planar structures. Polymers.

[47] Runnerstrom E.L., Llordés A., Lounis S.D., Milliron D.J. (2014). Nanostructured electrochromic smart windows: Traditional materials and NIR-selective plasmonic nanocrystals. Chem. Commun..

[48] Zhang Y., Pluchery O., Caillard L., Lamic-Humblot A.F., Casale S., Chabal Y.J., Salmeron M. (2015). Sensing the charge state of single gold nanoparticles via work function measurements. Nano Lett..

[49] Pluchery O., Zhang Y., Benbalagh R., Caillard L., Gallet J.J., Bournel F., Lamic-Humblot A.F., Salmeron M., Chabal Y.J., Rochet F. (2016). Static and dynamic electronic characterization of organic monolayers grafted on a silicon surface. Phys. Chem. Chem. Phys..

[50] Park Y., Choong V., Gao Y., Hsieh B.R., Tang C.W. (1996). Work function of indium tin oxide transparent conductor measured by photoelectron spectroscopy. Appl. Phys. Lett..

[51] Abdulrazzaq O., Bourdo S.E., Saini V., Watanabe F., Barnes B., Ghosh A., Biris A.S. (2015). Tuning the work function of polyaniline via camphorsulfonic acid: An X-ray photoelectron spectroscopy investigation. RSC Adv..

[52] Nardes A.M., Kemerink M., de Kok M.M., Vinken E., Maturova K., Janssen R.A.J. (2008). Conductivity, work function, and environmental stability of PEDOT:PSS thin films treated with sorbitol. Org. Electron..

[53] Abdellatif M.H., Ghosh S., Liakos I., Scarpellini A., Marras S., Diaspro A., Salerno M. (2016). Effect of nanoscale size and medium on metal work function in oleylamine-capped gold nanocrystals. J. Phys. Chem. Solids.

[54] Roduner E. (2006). Size matters: Why nanomaterials are different. Chem. Soc. Rev..

